# Gene Expression Profiling in the Type 1 Diabetes Rat Diaphragm

**DOI:** 10.1371/journal.pone.0007832

**Published:** 2009-11-13

**Authors:** Erik van Lunteren, Michelle Moyer

**Affiliations:** Pulmonary, Critical Care and Sleep Division, Department of Medicine, Louis Stokes Cleveland Department of Veterans Affairs Medical Center and Case Western Reserve University, Cleveland, Ohio, United States of America; Istituto Dermopatico dell'Immacolata, Italy

## Abstract

**Background:**

Respiratory muscle contractile performance is impaired by diabetes, mechanisms of which included altered carbohydrate and lipid metabolism, oxidative stress and changes in membrane electrophysiology. The present study examined to what extent these cellular perturbations involve changes in gene expression.

**Methodology/Principal Findings:**

Diaphragm muscle from streptozotocin-diabetic rats was analyzed with Affymetrix gene expression arrays. Diaphragm from diabetic rats had 105 genes with at least ±2-fold significantly changed expression (55 increased, 50 decreased), and these were assigned to gene ontology groups based on over-representation analysis using DAVID software. There was increased expression of genes involved in palmitoyl-CoA hydrolase activity (a component of lipid metabolism) (P = 0.037, n = 2 genes, fold change 4.2 to 27.5) and reduced expression of genes related to carbohydrate metabolism (P = 0.000061, n = 8 genes, fold change −2.0 to −8.5). Other gene ontology groups among upregulated genes were protein ubiquitination (P = 0.0053, n = 4, fold change 2.2 to 3.4), oxidoreductase activity (P = 0.024, n = 8, fold change 2.1 to 6.0), and morphogenesis (P = 0.012, n = 10, fold change 2.1 to 4.3). Other downregulated gene groups were extracellular region (including extracellular matrix and collagen) (P = 0.00032, n = 13, fold change −2.2 to −3.7) and organogenesis (P = 0.032, n = 7, fold change −2.1 to −3.7). Real-time PCR confirmed the directionality of changes in gene expression for 30 of 31 genes tested.

**Conclusions/Significance:**

These data indicate that in diaphragm muscle type 1 diabetes increases expression of genes involved in lipid energetics, oxidative stress and protein ubiquitination, decreases expression of genes involved in carbohydrate metabolism, and has little effect on expression of ion channel genes. Reciprocal changes in expression of genes involved in carbohydrate and lipid metabolism may change the availability of energetic substrates and thereby directly modulate fatigue resistance, an important issue for a muscle like the diaphragm which needs to contract without rest for the entire lifetime of the organism.

## Introduction

Diabetes mellitus impairs respiratory muscle function in humans with type 1 diabetes, as evidenced by findings of reduced vital capacity, peak esophageal and transdiaphragmatic pressures, maximal voluntary ventilation, and ability of the respiratory muscles to maintain a target force over time [1]–[3]. Studies in animal models of type 1 diabetes have confirmed reduced strength and endurance in respiratory and other skeletal muscles [4]–[10]. Diabetes-induced skeletal muscle dysfunction likely contributes to the reduced exercise capacity of humans with both type 1 [11] and type 2 [12], [13] diabetes, and may account for a portion of the increased sensation of dyspnea in humans with diabetes when ventilation or respiratory efforts are increased [3], [14].

Several cellular mechanisms underlying these adverse contractile changes have been identified from biochemical and electrophysiological studies of diaphragm muscle in animal models of diabetes. A well-established alteration is a shift in cellular energetics away from carbohydrate metabolism [15]–[22] and towards lipid metabolism [22]–[25]. There is also evidence for increased oxidative stress [6]. In addition there are perturbations in muscle electrophysiological properties involving resting membrane potential and action potential [7], [26], [27]. The extent to which these events involve changes in gene expression, and whether there are also changes in gene expression involving additional processes implicated in diabetes-induced dysfunction in other tissues (eg. protein ubiquitination), has not been elucidated previously for the respiratory muscles.

Alterations in gene expression with diabetes are prominent in many tissues, including pancreas [28], kidney [29]–[31], liver [32], spleen [33], adipose tissue [32], eye [34], corpus cavernosum [35], heart [36]–[38] and limb skeletal muscle [32], [39]–[41], albeit with important differences among tissue types. Normal diaphragm and limb skeletal muscles differ considerably in their patterns of gene expression [42]. Furthermore, the diaphragm differs importantly from limb muscle with respect to the degree and nature of alterations in gene expression in response to other diseases [42]–[44]. Thus previous data on limb muscle gene expression responses to diabetes [32], [39]–[41] are unlikely to apply directly to the diaphragm, in particular with respect to which specific genes undergo altered expression, the magnitude of these expression changes, and whether there are changes in gene expression related to cellular energetics. The purpose of the present study was to examine global alterations in gene expression of the diaphragm muscle in response to type 1 diabetes mellitus. We hypothesized that there would in particular be alterations in gene expression involving lipid and carbohydrate metabolism, oxidative stress and membranous ion channels.

## Methods

All studies were approved by the institutional animal care and use committee and conformed with NIH guidelines for animal care. Studies were performed on six male Wistar rats obtained from Charles River Laboratories (Wilmington, MA); cardiac data from these animals have been reported previously [38]. At an age of eight weeks, half of the animals were injected intraperitoneally with streptozotocin 60 mg/kg dissolved in sodium citrate buffer, and the other half with buffer alone. Four weeks later they were well-anesthetized with a mixture of intraperitoneal ketamine, xylazine and acepromazine following an all-night fast. Blood obtained from the tail was analyzed for glucose using a glucometer (Lifescan, Milpitas, CA). The entire costal diaphragm muscle was removed surgically, placed in RNAlater, and stored at −80°C; only the left hemidiaphragm was used as this provided sufficient tissue for RNA isolation. At the time of muscle removal, fasting blood glucose values were 70±4 mg/dl (range 62–76) for the normal animals, and 279±15 mg/dl (range 254–306) for the diabetic animals (P<0.001 by unpaired t test). The normal animals all gained weight during the four weeks following buffer injection (average 94±6 grams) whereas the diabetic animals lost 4–5% of their weight during the four weeks following streptozotocin injection (average 11±1 grams). Animals were not treated with insulin or oral hypoglycemics because the purpose of the study was to determine the effects of diabetes on diaphragm gene expression rather than the extent to which treatment of diabetes would attenuate the changes.

Gene expression array studies were performed in a manner similar to that described previously [38], [45], [46]. Total RNA was extracted using Trizol (GibcoBRL, Rockville, MD), and the RNA pellets were resuspended at 1 µg RNA/µl DEPC-treated water. This was followed by a cleanup protocol with a Qiagen (Valencia, CA) RNeasy Total RNA mini kit. Total RNA was prepared for use on Affymetrix (Santa Clara, CA) microarrays, according to the directions from the manufacturer. Briefly, 8 µg of RNA was used in a reverse transcription reaction (SuperScript II; Life Technologies, Rockville, MD) to generate first strand cDNA. After second strand synthesis, double strand cDNA was used in an *in vitro* transcription reaction to generate biotinylated cRNA. This was purified and fragmented, following which 15 µg of biotin-labeled cRNA was used in a 300 µl hybridization cocktail which included spiked transcript controls. 200 µl of cocktail was loaded onto Affymetrix RAE 230A microarrays (Santa Clara, CA) and hybridized for 16 hr at 45°C with agitation. Standard post-hybridization washes and double-stain protocols used an Affymetrix GeneChip Fluidics Station 400. Arrays were scanned using a Hewlett Packard Gene Array scanner, and analyzed with Affymetrix MAS 5.0 software. The data have been deposited in NCBIs Gene Expression Omnibus (GEO, http://www.ncbi.nlm.nih.gov/geo/) and assigned Series accession number GSE4653.

Gene expression array studies are critically dependent on high quality RNA both before and after the IVT amplification. Quality control values from the present study are as follows: 260/280 nm optical density prior to IVT of 1.97 to 2.18, RIN number from Agilent Bioanalyzer of 8.9 to 9.2, yield of RNA from IVT reaction of 71 to 129 micrograms, percent present calls on each array of 56 to 64 percent, betaActin 3′/M ratio of 1.4 to 2.3, betaActin 3′/5′ ratio of 1 to 1.4, GADPH 3′/M ratio of 1 to 1.4, and GAPDH 3′/5′ ratio of 1.1 to 1.8.

Statistical analysis was done with Bayesian analysis of variance for microarrays (BAM), using BAMarray software (http://www.bamarray.com) [47]. BAM balances the number of false detections against false non-detections by means of a special type of inferential regularization (i.e. borrowing strength across the data). Genes identified by BAM as having significantly changed expression were then further selected based on consistent and appropriate present and absent calls in all three samples of each group per Affymetrix software (marginal calls were accepted only if that gene was called present for all other samples in that group). Subsequently signals were averaged for muscle from the non-diabetic and from diabetic animals, and fold changes were calculated based on average values from each group. Analysis focused on genes whose expression changed at least ±2 fold in diabetic compared with control muscle, unless indicated otherwise. To assign biological meaning to the group of genes with changed expression, the subset of genes which met the above criteria was analyzed with the Gene Ontology (GO) classification system, using DAVID software (http://apps1.niaid.nih.gov/david/) [48]. Over-representation of genes with altered expression within specific GO categories was determined using the one-tailed Fisher exact probability modified by the addition of a jackknifing procedure, which penalizes the significance of categories with very few (eg. one or two) genes and favors more robust categories with larger numbers of genes [49].

Real-time PCR (RT-PCR) was used to confirm changes in gene expression as described previously [38], [45], [46]. Testing was done using the same tissue that had been used for gene expression arrays, and was performed for 31 genes. Genes which underwent PCR testing were chosen from specific, statistically over-represented, GO groups: palmitoyl-CoA hydrolase activity, protein ubiquitination, oxidoreductase activity, carbohydrate and alcohol metabolism, extracellular matrix and collagen. One gene not from one of these groups was also chosen for PCR testing due to an important role in muscle contraction (parvalbumin). An Applied Biosystems ABI 7900HT unit with automation attachment (Foster City, CA) was used for real-time PCR. This unit is capable of collecting spectral data at multiple points during a PCR run. To execute the first step and make archive cDNA, 3 µg of total RNA were reverse transcribed in a 100 µl reaction using Applied Biosystems enzymes and reagents in accordance with the manufacturer's protocols. RNA samples were accurately quantitated using a Nanodrop Technologies ND-1000 spectrophotometer (Wilmington, DE). Equal amounts of total RNA were reverse transcribed and then used in PCR amplifications. β-Actin had very little variation in expression across the sample set and therefore was chosen as the endogenous control. Since many of the target genes of interest were signaling molecules and likely to be expressed at low levels, we opted for a low dilution factor so as to create an environment more conducive to obtaining reliable results. The cDNA reaction from above was diluted by a factor of 10. For the PCR step, 9 µl of this diluted cDNA were used for each of three replicate 15 µl-reactions carried out in a 384 well plate. The genes tested and their respective Applied Biosystems rat assay catalog numbers are listed in Appendix S1. Standard PCR conditions were used for the Applied Biosystems assays: 50°C for 2 min, followed by 95°C for 10 min, followed by 40 cycles of 95°C for 15 sec alternating with 60°C for 1 min each. rtPCR analysis was similar to our previous study [38], [45], [46]. Values for RNA abundance were normalized for each gene with respect to the endogenous control in that sample (β-Actin), mean values for fold changes were calculated for each gene, and statistical testing was performed with the unpaired t-test.

## Results

There were 55 genes with significantly increased expression and 50 genes with significantly reduced expression in diabetic compared with normal diaphragm, using the cut-off of at least a ±2-fold changed expression in addition to consistent present calls by Affymetrix software and statistical signficance by BAM. A complete list of these genes, including mean fold change values for each gene, is provided in Appendix S2. Using lower fold-change thresholds also resulted in findings of roughly equal numbers of genes with increased and decreased expression, eg. 144 increases versus 136 decreases using a ±1.5-fold change threshold. Classification of genes by Gene Ontology (GO) groups and statistical testing of over-representation among GO groups was done separately for genes with increased and reduced expression.

### Genes with at Least 2-Fold Increased Expression in Diabetic Diaphragm

Among the 55 genes with at least 2-fold increased expression, assignment to GO groups was possible for 43 using the biological function classification, 41 using the molecular function classification, and 31 using the cellular constituent classification. The GO terms with over-representation among these genes are indicated in Table 1. The largest group with respect to both degree of significance and number of genes (n = 30) was the molecular function term catalytic activity. The other group with a large number of genes (n = 23) was the cellular constituent term intracellular. Four more specific groups each with smaller numbers of genes were found as well. The first group, palmitoyl-CoA hydrolase activity (a molecular function term), contained two genes. The second group was comprised of four genes related to ubiquitination and ubiquitin-ligase actvity, which appeared under the biological process, molecular function and cellular constituent classifications. The third was a group of eight genes relating to the molecular function term oxidoreductase activity. The fourth group was comprised of a number of different GO biological process terms relating to development, morphogenesis and organogenesis, with a total of ten genes. The genes with increased expression which comprised some of the more specific GO terms are listed in Table 2.

**Table 1:** Gene ontology groups with significant over-representation among genes with ≥2-fold increased expression in diabetic.

GO Classification	Specific GO Term	Number of Genes	P Value
Biological Process	Protein Ubiquitination	4	0.0053
	Morphogenesis	10	0.012
	Bone Mineralization	3	0.012
	Bone Remodelling	3	0.035
	Organogenesis	8	0.047
	Skeletal Development	3	0.048
	Organ Development	8	0.048
Molecular Function	Catalytic Activity	30	0.000034
	Ligase Activity, Forming Carbon-Nitrogen Bonds	5	0.001
	Ubiquitin-Protein Ligase Actvitity	4	0.0038
	Ligase Actvitity	5	0.0054
	Oxidoreductase Activity	8	0.024
	Palmitoyl-CoA Hydrolase Activity	2	0.037
Cellular Constituent	Ubiquitin Ligase Complex	4	0.0033
	Intracellular	23	0.017

P values reflect statistical significance of each GO term being over-represented among genes with increased expression.

**Table 2:** Genes with ≥2-fold increased expression in diabetic diaphragm assigned to over-represented gene ontology groups.

Gene Name	Gene Symbol	GeneID	Fold Change
*Palmitoyl-CoA Hydrolase Activity*			
cytosolic acyl-CoA thioesterase 1	*Cte1*	50559	27.5
mitochondrial acyl-CoA thioesterase 1	*Mte1*	192272	4.2
*Protein Ubiquitination*			
tripartite motif-containing 63	*Trim63*	140939	3.4
F-box only protein 32	*Fbxo32*	171043	3.1
ring finger protein 39	*Rnf39*	171387	2.6
casitas B-lineage lymphoma b	*Cblb*	171136	2.2
*Oxidoreductase Activity*			
cytochrome P450, family 2, subfamily e, polypeptide 1	*Cyp2e1*	25086	6.0
flavin containing monooxygenase 3	*Fmo3*	84493	2.9
crystallin, lamda 1	*Cryl1*	290277	2.4
lysyl oxidase	*Lox*	24914	2.3
ceruloplasmin	*Cp*	24268	2.2
2,4-dienoyl CoA reductase 1, mitochondrial	*Decr1*	117543	2.2
aldehyde oxidase 1	*Aox1*	54349	2.1
P450 (cytochrome) oxidoreductase	*Por*	29441	2.1
*Morphogenesis, Organogenesis, and Organ Development*			
endothelial cell-specific molecule 1	*Esm1*	64536	4.3
pregnancy-induced growth inhibitor	*Okl38*	171493	4.0
musculoskeletal, embryonic nuclear protein 1	*Mustn1*	290553	3.2
osteomodulin	*Omd*	83717	3.2
secreted phosphoprotein 1	*Spp1*	25353	2.9
serine (or cysteine) proteinase inhibitor, clade E, member 1	*Serpine1*	24617	2.8
ring finger protein 39	*Rnf39*	171387	2.6
v-maf musculoaponeurotic fibrosarcoma oncogene family, protein K (avian)	*Mafk*	246760	2.5
glycoprotein (transmembrane) nmb	*Gpnmb*	113955	2.5
dual-specificity tyrosine-(Y)-phosphorylation regulated kinase 1A	*Dyrk1a*	25255	2.1

### Genes with at Least 2-Fold Reduced Expression in Diabetic Diaphragm

Among the 50 genes with at least 2-fold reduced expression, assignment to GO groups was possible for 33 using the biological function classification, 41 using the molecular function classification, and 29 using the cellular constituent classification. The GO terms with over-representation among these genes are indicated in Table 3. There were a number of specific GO terms that revolved around the biological process theme of carbohydrate metabolism; this included a total of eight genes, with considerable overlap among these specific GO terms in constituent genes. A second prominent group was the cellular constituent term extracellular region containing 13 genes, which included more specific GO terms such as extracellular matrix and collagen. The third group was related to various molecular function terms dealing with binding (12 genes). Finally there were seven genes related to GO biological process themes organogenesis and organ development. The genes with reduced expression which comprise three of the more specific GO terms or groups of related GO terms are listed in Table 4.

**Table 3:** Gene ontology groups with significant over-representation among genes with ≥2-fold reduced expression in diabetic.

GO Classification	Specific GO Term	Number of Genes	P Value
Biological Process	Carbohydrate Metabolism	8	0.000061
	Alcohol Metabolism	6	0.0077
	Glucose Metabolism	5	0.0036
	Alcohol Catabolism	4	0.0038
	Cellular Carbohydrate Catabolism	4	0.0042
	Cellular Carbohydrate Metabolism	5	0.005
	Hexose Metabolism	5	0.0059
	Monosaccharide Metabolism	5	0.0059
	Glucose Catabolism	4	0.011
	Hexose Catabolism	4	0.011
	Monosaccharide Catabolism	4	0.011
	Glycolysis	4	0.02
	Organogenesis	7	0.032
	Organ Development	7	0.033
	Generation of Precursor Metabolites and Energy	6	0.048
Molecular Function	Extracellular Matrix Structural Constituent	5	0.00001
	Calcium Ion Binding	10	0.00003
	Ion Binding	12	0.0013
	Metal Ion Binding	12	0.0013
	Cation Binding	10	0.0072
Cellular Constituent	Collagen	4	0.00014
	Extracellular Region	13	0.00032
	Fibrillar Collagen	3	0.00033
	Extracellular Matrix (Sensu Metazoa)	6	0.0026
	Extracellular Matrix	6	0.0027
	Extracellular Space	8	0.011
	Collagen Type V	2	0.018
	Organelle Lumen	2	0.046

P values reflect statistical significance of each GO term being over-represented.

**Table 4:** Genes with ≥2-fold reduced expression in diabetic diaphragm assigned to over-represented gene ontology groups.

Gene Name	Gene Symbol	GeneID	Fold Change
*Carbohydrate and Alcohol Metabolism*			
neuraminidase 2	*Neu2*	29204	−8.5
phosphofructokinase, liver, B-type	*Pfkl*	25741	−3.7
solute carrier family 37 (glycerol-6-phosphate transporter), member 4	*Slc37a4*	29573	−2.5
glycerol-3-phosphate dehydrogenase 2	*Gpd2*	25062	−2.3
amylase 1, salivary	*Amy1*	24203	−2.3
phosphoglycerate mutase 2	*Pgam2*	24959	−2.1
lactate dehydrogenase A	*Ldha*	24533	−2.0
phosphoglucomutase 1	*Pgm1*	24645	−2.0
dihydrolipoamide S-acetyltransferase (E2 component of pyruvate dehydrogenase complex)	*Dlat*	81654	−2.0
*Extracellular Matrix and Collagen*			
collagen, type III, alpha 1	*Col3a1*	84032	−3.7
collagen, type 1, alpha 1	*Col1a1*	29393	−3.5
procollagen, type I, alpha 2	*Col1a2*	84352	−3.2
fibrillin 1	*Fbn1*	83727	−2.8
secreted acidic cysteine rich glycoprotein	*Sparc*	24791	−2.7
collagen, type V, alpha 1	*Col5a1*	85490	−2.4
collagen, type V, alpha 3	*Col5a3*	60379	−2.2
*Organogenesis and Organ Development*			
collagen, type III, alpha 1	*Col3a1*	84032	−3.7
Parvalbumin	*Pvalb*	25269	−3.1
acyl-CoA synthetase long-chain family member 6	*Acsl6*	117243	−2.7
ret proto-oncogene	*Ret*	24716	−2.5
chemokine (C-X-C motif) ligand 12	*Cxcl12*	24772	−2.4
reticulon 4	*Rtn4*	83765	−2.4
dimethylarginine dimethylaminohydrolase 1	*Ddah1*	64157	−2.1

### Genes with Lesser Degrees of Altered Expression

To determine whether other GO groups with over-representation may be present in a larger group of genes with more modest changes in expression, the GO group assignment was reanalyzed using the 280 genes with at least ±1.5-fold changed expression. Most of the themes identified above in the smaller group of genes with ±2-fold changed expression were confirmed, and furthermore had larger numbers of constituent genes. Among genes with increased expression, the ubiquitin group increased in size to 5 genes (from 4), the oxidoreductase group to 14 genes (from 8), and the morphogenesis group to 19 genes (from 10). Among genes with reduced expression, the carbohydrate metabolism group grew to 13 genes (from 8), the extracellular matrix group grew to 11 genes (from 6), and the organogenesis group grew to 15 genes (from 7).

### Confirmation Studies

**Figure 1:**
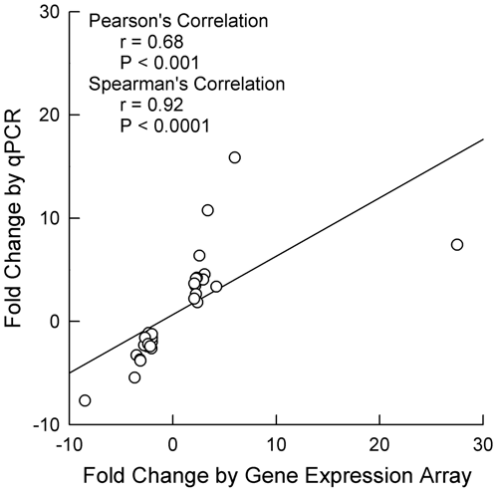
Relationship between fold changes in gene expression by expression array versus qPCR. Correlation coefficients are indicated for both Pearson's and Spearman's correlation methods.

To confirm changes in gene expression, high throughput RT-PCR was performed on a subset of 31 genes with at least ±2-fold changed expression (Table 5). Genes were chosen predominantly from the following specific GO groups with over-representation among genes with increased and decreased expression: palmitoyl-CoA hydrolase activity, protein ubiquitination, oxidoreductase activity, carbohydrate/alchohol metabolism, and collagen/extracellular matrix. Among the 31 genes listed in Tables 2 and 4, the direction of changes determined by PCR were in the same direction as that determined by expression arrays in 30 cases (97%). There was a good and statistically significant correlation between the magnitude of altered expression measured by gene expression array and that measured by RT-PCR for these genes (Figure 1). In most instances, the changes measured by PCR were statistically significant (Table 5).

**Table 5:** Changes in gene expression measured by real-time PCR.

Gene Symbol	Fold Change by Microarray	Fold Change by PCR	P Value by PCR
*Cyp2e1*	6.0	15.9	0.002
*Trim63*	3.4	10.7	0.004
*Cte1*	27.5	7.4	0.032
*Rnf39*	2.6	6.4	0.009
*Fbxo32*	3.1	4.5	0.002
*Lox*	2.3	4.2	0.001
*Decr1*	2.2	4.1	0.007
*Fmo3*	2.9	4.1	0.006
*Aox1*	2.1	3.7	0.006
*Cblb*	2.2	3.5	0.006
*Mte1*	4.2	3.4	0.003
*Cp*	2.2	2.7	0.117
*Por*	2.1	2.2	0.017
*Cryl1*	2.4	1.9	0.172
*Pfkl*	−3.7	1.2	0.396
*Gpd2*	−2.3	−1.1	0.488
*Dlat*	−2.0	−1.2	0.128
*Amy1*	−2.3	−1.4	0.233
*Sparc*	−2.7	−1.6	0.022
*Pgm1*	−2.0	−1.6	0.012
*Ldha*	−2.0	−2.0	0.022
*Col5a1*	−2.4	−2.2	<.001
*Fbn1*	−2.8	−2.3	0.002
*Col5a3*	−2.2	−2.4	0.001
*Slc37a4*	−2.5	−2.4	0.002
*Pgam2*	−2.1	−2.6	0.003
*Col1a1*	−3.5	−3.3	0.001
*Col1a2*	−3.2	−3.7	0.022
*Pvalb*	−3.1	−3.8	0.014
*Col3a1*	−3.7	−5.4	<.001
*Neu2*	−8.5	−7.7	0.005

## Discussion

The present study found that changes in diaphragm gene expression in a model of type 1 diabetes are divided almost equally between increases and decreases. There was a small shift towards genes involved in lipid metabolism, and a large shift away from genes involved in carbohydrate metabolism, supporting our hypothesis of diabetes altering the pattern of gene expression related to energy metabolism. There was also increased expression of genes related to oxidative stress (oxidoreductase activity), a processes implicated previously in diabetes-induced diaphragm dysfunction, as well as protein ubiquitination. On the other hand expression of genes involved in collagen and extracellular matrix formation was reduced, which in previous studies on limb muscle has been attributed to muscle atrophy. Finally there was both increased and decreased expression of genes related to morphogenesis, suggesting that there may be tissue remodelling occuring in conjunction with muscle atrophy. The following discussion will focus on type 1 diabetes.

### Palmitoyl-CoA Hydrolase Activity

The two genes with increased expression that belonged to the GO group palmitoyl-CoA hydrolase activity are involved in the reaction palmitoyl-CoA+H2O = CoA+palmitate, an important step in lipid metabolism (specifically long chain fatty acid metabolism). *Cte1* and *Mte1* are the cytosolic and mitochondrial forms respectively. Type 1 diabetes causes a 26-fold increased *Cte1* expression in rat heart [38], [50] as well as upregulated expression of *Mte1* in rodent limb skeletal and cardiac muscle [38], [51]. Durgan et al. [50] found that fatty acids upregulate cardiac *Cte1* expression, and that this involved regulatory roles for peroxisome proliferator-activated receptor-α but not insulin. In the present study there was another gene with 6-fold increased expression related to fatty acid metabolism (albeit not assigned to an over-represented GO group), namely *Hmgcs2*. The mitochondrial enzyme catalyzes a step in ketone-body synthesis, condensing acetyl-CoA with acetoacetyl-CoA to form HMG-CoA; expression is known to increase in the face of starvation [52]. High degrees of ketone body production are hallmarks of both diabetic ketoacidosis and starvation ketosis.

### Carbohydrate and Alcohol Metabolism

Nine genes with reduced expression were assigned to GO groups related to various aspects of carbohydrate and alcohol metabolism (*Neu2*, *Pfk1*, *Slc37a4*, *Gpd2*, *Amy1*, *Pgam2*, *Ldha*, *Pgm1*, *Dlat*). Among these, *Pfk1* catalyzes the reaction ATP+D-fructose 6-phosphate = ADP+D-fructose 1,6-bisphosphate, and is a key regulatory enzyme in glycolysis. *Slc37a4* is involved in the transmembrane transport of glycerol-6-phosphate; deficiency of this enzyme in humans has been implicated as the cause of glycogen storage disease type 1b [53] (in contrast to the more common type 1a variant which is caused by deficiency of glucose-6-phosphatase). *Pgam2* catalyzes the interconversion of 3- and 2-phosphoglycerate, and is involved in glycolysis-gluconeogenesis. *Ldha* catalyzes the reaction (S)-lactate+NAD+ = pyruvate+NADH+H+, and thus is involved in pyruvate metabolism as well as glycolysis-gluconeogenesis. *Pgm1* connects glycolysis and glycogen metabolism by interconverting glucose-1-phosphate and glucose-6-phosphate. *Dlat* is one of three components of the pyruvate dehydrogenase complex, which catalyzes the overall conversion of pyruvate to acetyl-CoA and CO_2_. Several of these genes have been shown previously to have altered expression in type 1 diabetes, including decreased expression of *Pgam* and *Ldha* in skeletal muscle [39] but increased expression of *Ldha* in pancreas [54].

### Protein Ubiquitination

There were four genes with increased expression related to ubiquitin, *Trim 63*, *Fbxo32*, *Rnf 39*, and *Cblb*. Ubiquitin ligases catalyze the reaction ATP+ubiquitin+protein lysine = AMP+diphosphate+protein N-ubiquityllysine. These enzymes catalyze the mediation of substrate recognition in ubiquitin-mediated protein degradation, which involves adding one or more ubiquitin moieties to the protein. Ubiquitin-mediated protein degradation has been implicated in the pathophysiology of type 1 diabetes [39], [55]–[57] as well as other catabolic states which cause muscle atrophy [39], [55]. Regarding individual genes within this group, increased expression of both *Fbxo32* (also known as *atrogin-1* and *MAFbx*) and *Trim63* (also known as *Murf1*) have been demonstrated in diabetic limb muscle [39], [58]. Trim63 catalyzes ubiquitylation of the contractile protein troponin I through a RING finger-dependent mechanism [59]. *Cblb* is a major susceptibility gene for type 1 diabetes in the Komeda diabetes-prone rat [60].

### Oxidoreductase Activity

There were eight genes with increased expression in the oxidoreductase GO group (*Cyp2e1*, *Fmo3*, *Cryl1*, *Lox*, *CP*, *Decr1*, *Aox1*, *Por*). Oxidoreductases catalyze an oxidation-reduction (redox) reaction, in which one substrate acts as a hydrogen or electron donor and becomes oxidized and the other acts as a hydrogen or electron recipient and becomes reduced. There is considerable evidence implicating oxidative stress in the pathophysiology of rodent experimental as well as human clinical type 1 diabetes [61]. Previous studies have found increased expression with type 1 diabetes of *Cyp2e1* in lymphocytes and heart and *Cp* in corpus cavernosum, whereas *Lox* undergoes reduced expression in cavernosum [35], [38], [62]. *Cyp2e1* is of particular interest in that protein levels increase in pancreas, liver, kidney and brain of type 1 diabetic rats in parallel to reactive oxygen species production and lipid peroxidation; furthermore overexpression of Cyp2e1 augments both glutathione S-transferase A4-4 levels and mitochondrial reactive oxygen species [61].

### Collagen and Extracelluar Matrix

There were seven genes with reduced expression in diabetic diaphragm muscle related to collagen and extracellular matrix (*Fbn1*, *Sparc*, and five collagen genes). Expression of *Fbn1*, *Col1a1* and *Col3a1* is reduced by type 1 diabetes in skeletal muscle [39], expression of these three genes as well as *Col5a3*, *Col5a1* and *Sparc* is reduced by type 1 diabetes in corpus cavernosum [35], *Sparc* expression is reduced in type 1 diabetic kidneys [63], and *Fbn1* and *Col3a1* expression is reduced in type 1 diabetic heart muscle [38].

### Morphogenesis, Organogenesis and Organ Development

There were ten genes with increased expression (*Esm1*, *Okl38*, *Mustn1*, *Omd*, *Spp1*, *Serpine1*, *Rnf39*, *Mafk*, *Gpnmb*, *Dyrk1a*) and seven genes with reduced expression (*Col3a1*, *Pvalb*, *Acsl6*, *Ret*, *Cxcl12*, *Rtn4*, *Ddah1*) related to morphogenesis, organogenesis and organ development, a group involved in the formation and organization of tissue and organ structure. No obvious distinguishing features could be discerned between constituent genes with increased compared with decreased expression, except that the former included three genes assigned to the GO group skeletal development (*Omd*, *Spp1*, *Gpnmb*). Among genes with reduced expression, *Pvalb* is of particular interest in that it binds calcium thereby playing an important role in skeletal muscle relaxation. Expression of *Pvalb* is decreased in skeletal muscle of streptozotocin-diabetic rats [39].

### Previous Gene Expression Array Studies of Limb Muscles

Several previous gene array studies have examined limb muscle gene expression responses to diabetes. Most are of type 2 diabetes [32], [40], [41] and will not be described here. Lecker et al. [39], on the other hand, studied streptozotocin-induced type 1 diabetes, similar to the present study. However their interest was to examine skeletal muscle atrophy, and hence they focused on limb muscle genes with altered expression that were common among four models: diabetes, cancer, renal failure and fasting. Gene groups with increased expression included those related to protein degradation and ubiquitin as well as transcription and translation. Reduced expression was found for groups of genes related to carbohydrate metabolism, ATP synthesis, and extracellular matrix proteins. Not described in any of the above four studies were changed expression of genes related to two other groups identified in the present study, oxidoreductase activity and morphogenesis/organogenesis. Thus the present study demonstrates a number of gene groups with altered expression in diaphragm that were previously identified as being affected in diabetic limb muscle. It also extends previous limb muscle findings by identifying two other gene groups whose expression in diaphragm is modulated by diabetes. One of these groups is related to oxidative stress, which based on numerous studies in other tissues is felt to be an important contributor to end-organ damage in diabetes [61].

### Relationship of Changes in Gene Expression to Cellular Function

Alterations in gene expression do not always parallel changes in protein levels or cellular function. However, for the presently demonstrated changes in gene expression there are data from other studies suggesting that at least some of the changes in gene expression correspond to post-transcriptional events that have been described previously [6], [8], [15]–[27]. First, there is extensive biochemical literature indicating that diabetes results in a shift in cellular energetics away from carbohydrate and towards lipid metabolism. Regarding the former, diabetic diaphragm has reduced uptake and phosphorylation of glucose, phosphorylation of fructose-6-phosphate, glycolysis, oxidation of pyruvate and acetate, uptake of acetoacete, production of glycogen, the proportion of the active complex of pyruvate dehydrogenase, and activities of hexokinase, phosphorylase and phosphofructokinase [15]–[22]. Regarding the latter, diabetic diaphragm has increased fat metabolism, uptake and oxidation of free fatty acids, output of glycerol (reflective of lipolysis), capacity for mobilization of intracellular lipids, and intracellular concentrations of triglycerides, free fatty acid and long-chain fatty acyl-CoA [22]–[25]. Thus the present findings indicate that alterations in gene expression contribute to the shift in cellular energetic patterns, and furthermore elucidate which specific lipid (*Cte1, Mte1*) and carbohydrate (*Neu2, Pfkl, Slc37a4, Gpd2, Amy1, Pgam2, Ldha, Pgm1, Dlat*) metabolism genes are involved.

Second, there is at least one previous functional study implicating heightened oxidative stress contributes to the impairment of diaphragm contractile performance resulting from diabetes. Hida et al. [6] administered N-acetylcysteine to rats with streptozotocin-induced diabetes, and found an attenuation of diabetes-induced contractile dysfunction. The present data thus provide further support for the role of oxidative stress in diabetes-induced diaphragm dysfunction by identifying eight genes related to oxidoreductase activity (*Cyp2e1, Fmo3, Cryl1, Lox, Cp, Decr1, Aox1, Por*) with increased expression in diabetic compared with normal diaphragm.

In contrast, it appears that diabetes-induced electrophysiological changes in diaphragm muscle can not be explained by changes in gene expression. Previously described diaphragm electrophysiological alterations include depolarization of resting membrane potential, shortening of action potential duration and reduced action potential area in an animal model of type 1 diabetes [26], and increased action potential height in a model of type 2 diabetes [27]. These electrophysiological parameters are regulated predominantly by Na+, K+ and Cl- channels and transporters, and no changes in expression exceeding ±2-fold of relevant genes were found in the present study.

### PCR Verification of Gene Expression Array Findings

Of the 31 genes which underwent PCR testing, for one gene the altered expression was in opposite directions with the two techniques, and for five genes the PCR data was not statistically significant although the direction of the changes paralleled that of the array data. Among the latter group of five genes, the lack of statistical significance for four genes (those with P values in the 0.05 to 0.25 range) most likely was due to the sample size, as the t-test does not perform real well with modest sample sizes (in contrast to BAM, used for statistical testing of the the array data, which borrows strength across the entire data set and thus is more robust with modest sample sizes). PCR data for the fifth gene had a much higher P value (0.488), and most likely the lack of significance would have persisted even with larger sample sizes. The presence of a small number of false positive findings is typical for gene expression array technology due to the fact that expression of well over >10,000 genes is being tested simultaneously.

### Methodological Issues

The present study used streptozotocin to produce diabetes. The major effect of streptozotocin is on the pancreatic islet cells, and thus in this model changes in other organs are believed to be due predominantly to the diabetes and not the streptozotocin – however it is certainly possible that some of the changes found in the present study could be due to the acute toxic effect of the streptozotocin. The present study used a single dose of streptozotocin rather than repeated low doses, which is consistent with previous studies of diaphragm muscle in streptozotocin-induced diabetes [6]–[9], [16], [17], [21], [25], [38] and previous gene expression array studies of other organ systems in streptozotocin-induced diabetes [34]–[37].

The confirmatory PCR studies were performed on tissue from the same animals used for the gene expression array studies. Among fifteen previous gene expression array studies of diabetes [28]–[41], [64], twelve reported PCR data. Nine of the twelve performed PCR on tissue from the same animals or humans as the gene expression arrays, two studies performed PCR on tissue from separate subjects, and one study performed PCR on 11 genes using tissue from the same animals and on 3 genes using different animals. Thus the majority of PCR data from previous gene expression array studies of diabetes is derived from the same animals or humans that were used for the expression arrays. In the present study we therefore performed PCR studies in the same animals as had been used in array studies in order to make our study more directly comparable to previous work in this area.

### Conclusions

In conclusion the present study found that diaphragm muscle gene expression was modified in several important areas of cellular function and structure in a model of type 1 diabetes. Reciprocal changes in expression of genes involved in carbohydrate and lipid metabolism may change the availability of energetic substrates and thereby directly modulate fatigue resistance, an important issue for a muscle like the diaphragm which needs to contract without rest for the entire lifetime of the organism. Diabetes is well-established to reduce cardiac muscle carbohydrate uptake and oxidation and increase fatty acid metabolism, which is believed to directly impair contractility and increase the severity of ischemic injury [65]. Interestingly, in type 1 diabetic heart there is more prominent increased expression of lipid metabolism genes than decreased expression of carbohydrate metabolism genes [36]–[38], whereas in the type 1 diabetic diaphragm the number of carbohydrate genes with decreased expression exceeds the number of lipid metabolism with increased expression. Upregulation of ubiquitin-related gene expression suggests a role for a specific pathway of protein degradation, one of which has been demonstrated to have as its target a member of the contractile apparatus. Augmented oxidoreductase gene expression suggests that oxidative stress may contribute to respiratory muscle dysfunction, as has been implicated with diabetes in other tissues [61]. Finally, altered expression of number of genes noted in the present study is also found in a number of conditions leading to muscle atrophy, which might contribute to respiratory muscle dysfunction in any state associated with cachexia. Thus the present data suggest the presence of several processes which contribute and possibly interact to impair diaphragm muscle contractile function in type 1 diabetes, several of which are potentially amenable to therapeutic interventions. A limitation of the present study is the small number of animals studied with its effects on the robustness of the findings. Future studies are needed to test the efficacy of interventions which specifically address the processes suggested by the present gene expression studies. One such study has already been performed, namely the effects of the anti-oxidant N-acetylcysteine [6], which did indeed attentuate the diabetes-induced contractile dysfunction. Other studies could include pharamacologic or dietary interventions which change cellular substrate utilization patterns or drugs which modulate ubiquitin-mediated protein degradation.

## Supporting Information

Appendix S1 Genes which underwent RT-PCR testing, and the respective Applied Biosystems rat assay catalog numbers.
(0.06 MB DOC)

Appendix S2 Complete list of genes with statistically significant changes of at least±2-fold in diabetic compared with normal diaphragm muscle.
(0.15 MB DOC)
