# Supplementary material for: Gene Expression Profiling in the Type 1 Diabetes Rat Diaphragm
Source: PLoS One. 2009 Nov 13;4(11):e7832. doi: 10.1371/journal.pone.0007832 (PMC2773011; doi:10.1371/journal.pone.0007832)
Supplement: Appendix S1 — Genes which underwent RT-PCR testing, and the respective Applied Biosystems rat assay catalog numbers. (0.06 MB DOC) [file pone.0007832.s001.doc]

| Gene Symbol | GeneID | Gene Name | Applied Biosystems Catalog Number |
| --- | --- | --- | --- |
| Amy1 | 24203 | amylase 1, salivary | Rn01522158_g1 |
| Aox1 | 54349 | aldehyde oxidase 1 | Rn00571242_m1 |
| Cblb | 171136 | Casitas B-lineage lymphoma b | Rn00709852_m1 |
| Col1a1 | 29393 | collagen, type 1, alpha 1 | Rn00801649_g1 |
| Col1a2 | 84352 | procollagen, type I, alpha 2 | Rn00584426_m1 |
| Col3a1 | 84032 | collagen, type III, alpha 1 | Rn01437683_m1 |
| Col5a1 | 85490 | Collagen, type V, alpha 1 | Rn00593170_m1 |
| Col5a3 | 60379 | collagen, type V, alpha 3 | Rn00572797_m1 |
| Cp | 24268 | ceruloplasmin | Rn00561049_m1 |
| Cryl1 | 290277 | crystallin, lamda 1 | Rn00598394_m1 |
| Cte1 | 50559 | cytosolic acyl-CoA thioesterase 1 | Rn01789864_s1 |
| Cyp2e1 | 25086 | cytochrome P450, family 2, subfamily e, polypeptide 1 | Rn00580624_m1 |
| Decr1 | 117543 | 2,4-dienoyl CoA reductase 1, mitochondrial | Rn00589420_m1 |
| Dlat | 81654 | dihydrolipoamide S-acetyltransferase (E2 component of pyruvate dehydrogenase complex) | Rn01766568_g1 |
| Fbn1 | 83727 | fibrillin 1 | Rn00582774_m1 |
| Fbxo32 | 171043 | F-box only protein 32 | Rn00591730_m1 |
| Fmo3 | 84493 | Flavin containing monooxygenase 3 | Rn00584825_m1 |
| Gpd2 | 25062 | Glycerol-3-phosphate dehydrogenase 2 | Rn00562472_m1 |
| Ldha | 24533 | lactate dehydrogenase A | Rn00820751_g1 |
| Lox | 24914 | lysyl oxidase | Rn00566984_m1 |
| Mte1 | 192272 | mitochondrial acyl-CoA thioesterase 1 | Rn02104940_s1 |
| Neu2 | 29204 | neuraminidase 2 | Rn00567505_m1 |
| Pfkl | 25741 | Phosphofructokinase, liver, B-type | Rn00566132_m1 |
| Pgam2 | 24959 | phosphoglycerate mutase 2 | Rn00569094_m1 |
| Pgm1 | 24645 | phosphoglucomutase 1 | Rn00566810_m1 |
| Por | 29441 | P450 (cytochrome) oxidoreductase | Rn00580820_m1 |
| Pvalb | 25269 | parvalbumin | Rn00574541_m1 |
| Rnf39 | 171387 | ring finger protein 39 | Rn00592689_m1 |
| Slc37a4 | 29573 | solute carrier family 37 (glycerol-6-phosphate transporter), member 4 | Rn00580926_m1 |
| Sparc | 24791 | secreted acidic cysteine rich glycoprotein | Rn00561955_m1 |
| Trim63 | 149039 | tripartite motif-containing 63 | Rn00590197_m1 |
