# Supplementary material for: Gene Expression Profiling in the Type 1 Diabetes Rat Diaphragm
Source: PLoS One. 2009 Nov 13;4(11):e7832. doi: 10.1371/journal.pone.0007832 (PMC2773011; doi:10.1371/journal.pone.0007832)
Supplement: Appendix S2 — Complete list of genes with statistically significant changes of at least±2-fold in diabetic compared with normal diaphragm muscle. (0.15 MB DOC) [file pone.0007832.s002.doc]

| Gene Title | Gene Symbol | GeneID | Fold Change |
| --- | --- | --- | --- |
| *Increases* |  |  |  |
| cytosolic acyl-CoA thioesterase 1 | *Cte1* | 50559 | 27.5 |
| growth arrest specific 5 | *Gas5* | 81714 | 6.8 |
| 3-hydroxy-3-methylglutaryl-Coenzyme A synthase 2 | *Hmgcs2* | 24450 | 6.0 |
| cytochrome P450, family 2, subfamily e, polypeptide 1 | *Cyp2e1* | 25086 | 6.0 |
| ankyrin repeat domain 1 (cardiac muscle) | *Ankrd1* | 27064 | 5.9 |
| endothelial cell-specific molecule 1 | *Esm1* | 64536 | 4.3 |
| mitochondrial acyl-CoA thioesterase 1 | *Mte1* | 192272 | 4.2 |
| pregnancy-induced growth inhibitor | *Okl38* | 171493 | 4.0 |
| eukaryotic translation initiation factor 4E binding protein 1 | *Eif4ebp1* | 116636 | 3.7 |
| adenosine monophosphate deaminase 3 | *Ampd3* | 25095 | 3.5 |
| tripartite motif-containing 63 | *Trim63* | 140939 | 3.4 |
| insulin induced gene 1 | *Insig1* | 64194 | 3.3 |
| CCAAT/enhancer binding protein (C/EBP), delta | *Cebpd* | 25695 | 3.3 |
| musculoskeletal, embryonic nuclear protein 1 | *Mustn1* | 290553 | 3.2 |
| osteomodulin | *Omd* | 83717 | 3.2 |
| F-box only protein 32 | *Fbxo32* | 171043 | 3.1 |
| RNA polymerase 1-4 | *Rpo1-4* | 83581 | 3.0 |
| flavin containing monooxygenase 3 | *Fmo3* | 84493 | 2.9 |
| phosphatidylinositol 3-kinase, C2 domain containing, gamma polypeptide | *Pik3c2g* | 116720 | 2.9 |
| secreted phosphoprotein 1 | *Spp1* | 25353 | 2.9 |
| serine (or cysteine) proteinase inhibitor, clade E, member 1 | *Serpine1* | 24617 | 2.8 |
| MAD homolog 7 (Drosophila) | *Madh7* | 81516 | 2.8 |
| ring finger protein 39 | *Rnf39* | 171387 | 2.6 |
| nuclear protein 1 | *Nupr1* | 113900 | 2.6 |
| v-maf musculoaponeurotic fibrosarcoma oncogene family, protein K (avian) | *Mafk* | 246760 | 2.5 |
| myelocytomatosis viral oncogene homolog (avian) | *Myc* | 24577 | 2.5 |
| glycoprotein (transmembrane) nmb | *Gpnmb* | 113955 | 2.5 |
| cathepsin L | *Ctsl* | 25697 | 2.4 |
| crystallin, lamda 1 | *Cryl1* | 290277 | 2.4 |
| lipocalin 7 | *Lcn7* | 94174 | 2.4 |
| lysyl oxidase | *Lox* | 24914 | 2.3 |
| glutamate cysteine ligase, modifier subunit | *Gclm* | 29739 | 2.3 |
| 2',5'-oligoadenylate synthetase 1, 40/46kDa | *Oas1* | 192281 | 2.3 |
| insulin-like growth factor 1 receptor | *Igf1r* | 25718 | 2.3 |
| ceruloplasmin | *Cp* | 24268 | 2.2 |
| 2,4-dienoyl CoA reductase 1, mitochondrial | *Decr1* | 117543 | 2.2 |
| choline kinase alpha | *Chka* | 29194 | 2.2 |
| coagulation factor 2 | *F2* | 29251 | 2.2 |
| synaptojanin 2 | *Synj2* | 84018 | 2.2 |
| serine racemase | *Srr* | 303306 | 2.2 |
| Hspb associated protein 1 | *Hspbap1* | 171460 | 2.2 |
| spermidine/spermine N1-acetyl transferase | *Sat* | 302642 | 2.2 |
| casitas B-lineage lymphoma b | *Cblb* | 171136 | 2.2 |
| dual-specificity tyrosine-(Y)-phosphorylation regulated kinase 1A | *Dyrk1a* | 25255 | 2.2 |
| transformation related protein 53 inducible nuclear protein 1 | *Trp53inp1* | 297822 | 2.1 |
| aldehyde oxidase 1 | *Aox1* | 54349 | 2.1 |
| sequestosome 1 | *Sqstm1* | 113894 | 2.1 |
| P450 (cytochrome) oxidoreductase | *Por* | 29441 | 2.1 |
| microtubule-associated protein 1b | *Map1b* | 29456 | 2.0 |
| proteasome (prosome, macropain) activator subunit 4 | *Psme4* | 498433 | 2.0 |
| myeloid differentiation primary response gene 116 | *Myd116* | 171071 | 2.0 |
| nucleoporin like 1 | *Nupl1* | 245922 | 2.0 |
| tubulin, beta, 2 | *Tubb2* | 296554 | 2.0 |
| fibrinogen-like 2 | *Fgl2* | 84586 | 2.0 |
| guanine nucleotide binding protein-like 3 (nucleolar) | *Gnl3* | 290556 | 2.0 |
|  |  |  |  |
| *Decreases* |  |  |  |
| myristoylated alanine rich protein kinase C substrate | *Marcks* | 25603 | -2.0 |
| phosphoserine aminotransferase 1 | *Psat1* | 293820 | -2.0 |
| dihydrolipoamide S-acetyltransferase (E2 component of pyruvate dehydrogenase complex) | *Dlat* | 81654 | -2.0 |
| phosphoglucomutase 1 | *Pgm1* | 24645 | -2.0 |
| lactate dehydrogenase A | *Ldha* | 24533 | -2.0 |
| dimethylarginine dimethylaminohydrolase 1 | *Ddah1* | 64157 | -2.1 |
| phosphoglycerate mutase 2 | *Pgam2* | 24959 | -2.1 |
| guanylate nucleotide binding protein 2 | *Gbp2* | 171164 | -2.1 |
| sushi-repeat-containing protein | *Srpx* | 64316 | -2.1 |
| adenylate cyclase 7 | *Adcy7* | 84420 | -2.1 |
| demethyl-Q 7 | *Coq7* | 25249 | -2.1 |
| evectin-1 | *Plekhb1* | 64471 | -2.2 |
| collagen, type V, alpha 3 | *Col5a3* | 60379 | -2.2 |
| ATP-binding cassette, sub-family D (ALD), member 2 | *Abcd2* | 84.56 | -2.2 |
| receptor (calcitonin) activity modifying protein 1 | *Ramp1* | 58965 | -2.2 |
| mitogen-activated protein kinase kinase 6 | *Map2k6* | 114495 | -2.2 |
| guanidinoacetate methyltransferase | *Gamt* | 25257 | -2.3 |
| amylase 1, salivary | *Amy1* | 24203 | -2.3 |
| follistatin-like 1 | *Fstl1* | 79210 | -2.3 |
| glycerol-3-phosphate dehydrogenase 2 | *Gpd2* | 25062 | -2.3 |
| collagen, type V, alpha 1 | *Col5a1* | 85490 | -2.4 |
| allograft inflammatory factor 1 | *Aif1* | 29427 | -2.4 |
| reticulon 4 | *Rtn4* | 83765 | -2.4 |
| carboxylesterase 3 | *Ces3* | 113902 | -2.4 |
| anti-Mullerian hormone type 2 receptor | *Amhr2* | 29530 | -2.4 |
| chemokine (C-X-C motif) ligand 12 | *Cxcl12* | 24772 | -2.4 |
| serine (or cysteine) proteinase inhibitor, clade H, member 1 | *Serpinh1* | 29345 | -2.5 |
| GTPase, IMAP family member 4 | *Gimap4* | 286938 | -2.5 |
| solute carrier family 37 (glycerol-6-phosphate transporter), member 4 | *Slc37a4* | 29573 | -2.5 |
| ret proto-oncogene | *Ret* | 24716 | -2.5 |
| follistatin | *Fst* | 24373 | -2.5 |
| acyl-CoA synthetase long-chain family member 6 | *Acsl6* | 117243 | -2.7 |
| secreted acidic cysteine rich glycoprotein | *Sparc* | 24791 | -2.7 |
| fibrillin 1 | *Fbn1* | 83727 | -2.8 |
| procollagen C-proteinase enhancer protein | *Pcolce* | 29569 | -2.9 |
| parvalbumin | *Pvalb* | 25269 | -3.1 |
| procollagen, type I, alpha 2 | *Col1a2* | 84352 | -3.2 |
| regucalcin | *Rgn* | 25106 | -3.3 |
| collagen, type 1, alpha 1 | *Col1a1* | 29393 | -3.5 |
| phosphofructokinase, liver, B-type | *Pfkl* | 25741 | -3.7 |
| collagen, type III, alpha 1 | *Col3a1* | 86032 | -3.7 |
| monocarboxylate transporter | *Slc16a3* | 80878 | -3.7 |
| aquaporin 4 | *Aqp4* | 25293 | -3.9 |
| carboxypeptidase A1 | *Cpa1* | 24269 | -4.4 |
| solute carrier family 25 (mitochondrial carrier, phosphate carrier), member 25 | *Slc25a25* | 246771 | -4.6 |
| neuronatin | *Nnat* | 94270 | -4.8 |
| apelin, AGTRL1 ligand | *Apln* | 58812 | -5.8 |
| neuraminidase 2 | *Neu2* | 29204 | -8.5 |
| retinol binding protein 4, plasma | *Rbp4* | 25703 | -8.6 |
| neuronal regeneration related protein | *Nrep* | 338475 | -12.9 |
